# Safety of adjuvant brachytherapy combined with external beam radiotherapy for proximal bile duct pR1 in perihilar cholangiocarcinoma: a prospective registration study

**DOI:** 10.1007/s00423-026-04180-6

**Published:** 2026-08-05

**Authors:** Hasan Ahmad Al-Saffar, Henrik Roed, Marianne Feen Rønjom, Britta Weber, Christoph Tschuor, Peter Nørgaard Larsen, Flemming Kjær-Kristoffersen, Morten Ladekarl, Hans-Christian Pommergaard

**Affiliations:** 1 Department of Digestive Diseases, Transplantation and General Surgery, Copenhagen University Hospital, Rigshospitalet, Copenhagen, 2100 Denmark; 2 Hepatic Malignancy Surgical Research Unit, Copenhagen University Hospital, Rigshospitalet, Copenhagen, 2100 Denmark; 3 Department of Oncology, Rigshospitalet, Copenhagen University Hospital, Copenhagen, 2100 Denmark; 4 Department of Oncology, Copenhagen University Hospital - Herlev and Gentofte, Herlev, 2730 Denmark; 5 Department of Oncology, Aarhus University Hospital, Aarhus, 8200 Denmark; 6 Danish Centre for Particle Therapy, Aarhus University Hospital, Aarhus, 8200 Denmark; 7 Department of Oncology, Clinical Cancer Research Centre, Aalborg University Hospital, Aalborg, 9000 Denmark; 8 Department of Clinical Medicine, Aalborg University, Aalborg, 9000 Denmark; 9 Department of Clinical Medicine, University of Copenhagen, Copenhagen, 2100 Denmark

**Keywords:** Brachytherapy, Adverse Events, Perihilar Cholangiocarcinoma, pR1

## Abstract

**Background:**

Microscopic residual disease (pR1) following resection of perihilar cholangiocarcinoma (pCCA) is associated with high recurrence rates. Adjuvant brachytherapy (BT) combined with external beam radiotherapy (EBRT) is proposed to improve local control; however, contemporary data on treatment-related toxicity are scarce.

**Methods:**

This prospective registration study included patients with pCCA and isolated pR1 at the proximal bile duct (PBD) margin treated with adjuvant BT and EBRT between January 2017 and December 2021. Primary endpoints were radiation-induced biliary strictures, and severe treatment-related adverse events (TRAEs). Secondary endpoints were overall survival (OS), disease-free survival (DFS) and local recurrence.

**Results:**

Eight patients were eligible of whom six received treatment. Median time from surgery to BT was 39.5 days, with a median follow up of 35.5 months. Early severe TRAEs were infrequent (*n* = 2). Radiation-induced biliary strictures occurred in four patients and late severe TRAEs were common (*n* = 16), predominatly by recurrent cholangitis, resulting in one death (*n* = 1). Four developed recurrence, all at distant sites and no local recurrences. Median DFS was 28.6 months (IQR 10.9–53.5) and median OS 34.3 months (IQR 19.4–39.9).

**Conclusion:**

Adjuvant BT combined with EBRT was associated with high rates of severe late TRAEs in resected pCCA. Despite absence of local recurrence, these findings raise concerns regarding routine use outside clinical trials.

**Supplementary Information:**

The online version contains supplementary material available at https://doi.org/10.1007/s00423-026-04180-6.

## Highlights

Adjuvant brachytherapy combined with external beam radiotherapy perihilar cholangiocarcinoma with pR1 is associated with high late toxicity.

Severe late TRAEs dominated by biliary complications.

Findings question routine use outside clinical trials.

**Supplementary Information:**

The online version contains supplementary material available at https://doi.org/10.1007/s00423-026-04180-6.

## Introduction

Perihilar cholangiocarcinoma (pCCA) is a rare but aggressive malignancy arising at the confluence of the right and left hepatic ducts [1]. Curatively intended resection offers a 5-year overall survival (OS) of 30 to 40% [2, 3] with local recurrence constituting the dominant pattern of failure [4]. Achieving microscopic radicality (pR0) is paramount for survival, but remains challenging with microscopic residual disease (pR1) rates at up to 50% [1, 5, 6]. This is often due to tumour proximity to essential hilar structures, or a limited future liver remnant (FLR) preventing major hepatectomy [2].

Inability to extend proximal bile duct (PBD) resection contributes to almost 20% of pR1 resections [7, 8]. This reason is a short PBD remnant where extended resection is balanced against preservation of an adequate FLR [9], or a safe hepaticojejunostomy [10]. Intraoperative re-resection of the PBD following frozen section (FS) analysis has yet to demonstrate a survival benefit [8]. With adjuvant chemotherapy showing limited impact on local disease control [11, 12], there is a need to investigate locoregional radiotherapeutic strategies for pR1 in PBD [13].

Brachytherapy (BT) combined with external beam radiotherapy (EBRT), is well established in palliative treatment for pCCA, delivering high-dose radiation directly to the bile duct (13–16). Its application in the adjuvant treatment setting, remains limited in the West. Available data are scarce, and derive from studies published three decades ago reporting conflicting survival outcomes [18–21] with substantial toxicity [19]. However, late toxicity burden of combined adjuvant BT with EBRT in the modern multimodal era remains poorly defined. Given the intrinsic radiosensitivity of biliary epithelium and altered postoperative anatomy, treatment-related morbidity may be considerable.

Following nationwide centralisation of curative surgery for pCCA in Denmark during the past decade, most patients are referred to Rigshospitalet, Copenhagen [22]. A protocol of combined adjuvant BT with EBRT was adopted for patients with isolated PBD pR1. This prospective registration study aims to evaluate safety, survival and feasibility of this treatment.

## Methods

### Study design and participants

This study included patients presenting with resectable pCCA from January 1, 2017 and December 31, 2021, who received adjuvant BT with EBRT for isolated PBD pR1. All patients were discussed at multidisciplinary team (MDT) meetings [23] attended by hepato-pancreato-biliary (HPB) surgeons, abdominal radiologists, HPB-pathologists, and oncologists. Resectability was based on the Bismuth-Corlette classification (I-IV) combined with the absence of: vascular invasion (≥ 180°) to the future liver remnant (FLR); regional lymph node metastasis; extrahepatic disease; an FLR-ratio < 30%; or underlying chronic liver disease (cirrhosis Child-Pugh B or C) [24]. Radiological assessment was performed using magnetic resonance imaging (MRI) and fluorodeoxyglucose positron emission tomography (FDG-PET). Preoperative induction chemotherapy was considered only in cases of vascular invasion involving the FLR, or regional lymph node metastases. Patients without the possibility of surgical exploration after induction chemotherapy, were referred to palliative chemotherapy or best supportive care. Reasons for cancellation of surgery included disease progression after neoadjuvant chemotherapy, physical deterioration prior to resection, complications related to biliary decompression, and tumour progress during waiting time.

The Danish Liver Cancer Group Database (DLGCD) is a prospective nationwide database maintained by the Danish Clinical Quality Program (SundK). It includes data on all patients with suspected pCCA discussed at the first MDT conference at Rigshospitalet, Copenhagen. Data were obtained on baseline characteristics including, age, gender, time interval between first MDT and resection, perioperative data, comprehensive comorbidity index (CCI) [25], time from resection to initiation of BT, oncological treatment, and recurrence. Of note, data on Eastern Cooperative Oncology Group Performance Status (ECOG-PS) [26], American Society of Anesthesiologists Physical Status (ASA) Classification [27], and body mass index (BMI), were retrieved at point of resection. Histopathological data were linked with the prospective Danish Pathology Data Bank (Patobank), while vital status was ascertained through linkage with the Danish Civil Registration System using unique personal identification numbers. Ethical approval was obtained from the Danish Health Authority in The Capital Region of Denmark (R-23057752), and individual informed consent was provided from patients or family members. The study followed the STROBE-guidelines [28].

### Preoperative procedures

Preoperative biliary drainage was routinely performed after MDT, using percutaneous transhepatic cholangiography (PTC). Portal vein embolization (PVE) was indicated when the FLR ratio was < 30%, or consisted of the left lateral Sects. [22, 29]. Details regarding local work-up and patient selection protocols, have been previously published [22, 29].

Combined adjuvant brachytherapy and external beam radiotherapy.

All patients with confirmed pCCA at histopathology and pR1 were evaluated at an MDT meeting following surgery. pR1 was defined as 0 mm margin clearance at either the PBD-, DBD-, or radial margin (RM) [30]. Patients with pR1 in the PBD margin, and no concurrent radial (RM)- and/or DBD margin pR1, were considered for combined BT and EBRT. This included patients undergoing extrahepatic bile duct resection with a positive intraoperative PBD frozen section who were unfit for major hepatectomy, or pancreaticoduodenectomy with concomitant hepatectomy. Of note, during the latter half of the inclusion period, updated RM assessment guidelines incorporating the circumferential margin, prompted revision of all included specimens [31]. All specimens were assessed by a consistent group of dedicated HPB-pathologists during the entire inclusion period. Other reasons for exclusion included other biliary tract carcinomas (BTC), liver transplantation, macroscopic residual disease (R2), ECOG-PS three or above, and postoperative bile leakage (International Study Group of Liver Surgery (ISGLS)) grade B or C [32].

Those referred to BT with EBRT retained the PTC-drain (minimum 10 French (Fr)) [30]. An 8 Fr blind-ended sheath was guided through the PTC by fluoroscopy and secured with tape, ensuring treatment positioning and measurements tailored to the hepaticojejunostomy. A 6 Fr Nucletron/Elekta marker catheter was subsequently passed through the entire blind-ended sheath, and sutured to the skin. Dose calculation was performed manually with Oncentra. A total dose of 20 Gy was delivered using pulse-dose-rate (PDR) BT with 1 Gy per hour prescribed 1 cm from the central axis of the catheter, 2 cm on both sides of the hepaticojejunostomy [14]. The PTC-drain was removed following completion of BT. Afterwards, external beam radiotherapy (EBRT) was administered within one week, at the hepatic hilum with 1.8 Gy per fraction, five fractions per week, to a total dose of 45 Gy [14]. Subsequently, adjuvant chemotherapy was offered at the local hospital to all patients. Reasons for not receiving adjuvant chemotherapy included ECOG-PS ≥ 3 following completion of radiotherapy, or patient refusal. Early patients received 4 cycles of gemcitabine (1000 mg/m²) and cisplatin (25 mg/m²), administered on days 1 and 8, followed by a two-week rest period, in accordance with the ESMO Clinical Practice Guideline [33]. Following publication of the BILCAP protocol in 2019, capecitabine was administered (1250 mg/m² twice daily) on days 1–14 of a 21-day cycle, for a total of eight cycles over six months [12]. Patients received routine clinical follow up with CA 19 − 9 monitoring at their local surgical outpatient clinics. Suspected recurrence prompted chest and abdominal CT imaging, with histological confirmation obtained by percutaneous biopsy or cholangioscopy-guided SpyBites [34].

### End-points

Primary endpoints were radiation-induced biliary strictures, and TRAEs following combined BT with EBRT. Radiation-induced biliary stricture was defined as extrahepatic bile duct narrowing at radiology with cholestasis and/or cholangitis, and exclusion of local recurrence (22,23). Each TRAE was graded 0 to 5 (CTCAE version 6.0) [35], and classified as early (≤ 90 days from BT initiation) or late (> 90 days from BT initiation) [35]. Secondary endpoints included local recurrence, OS and disease-free survival (DFS), both starting from the date of first MDT. Local recurrence was defined as any radiological sign of recurrence in the hepatic hilum [4]. OS was defined as months until date of death from any cause or last follow up. DFS was assessed until date of recurrence, death from any cause, or last follow up.

### Statistical analysis

Statistical analyses were performed using R 4.5.1. Demographic and clinicopathological variables were reported with missing data. Categorical variables were reported as numbers and percentages; continuous variables as medians with interquartile ranges (IQR) or means with standard deviations (SD). Median follow up was estimated using the reverse Kaplan–Meier method.

## Results

### Study group

**Fig. 1:**
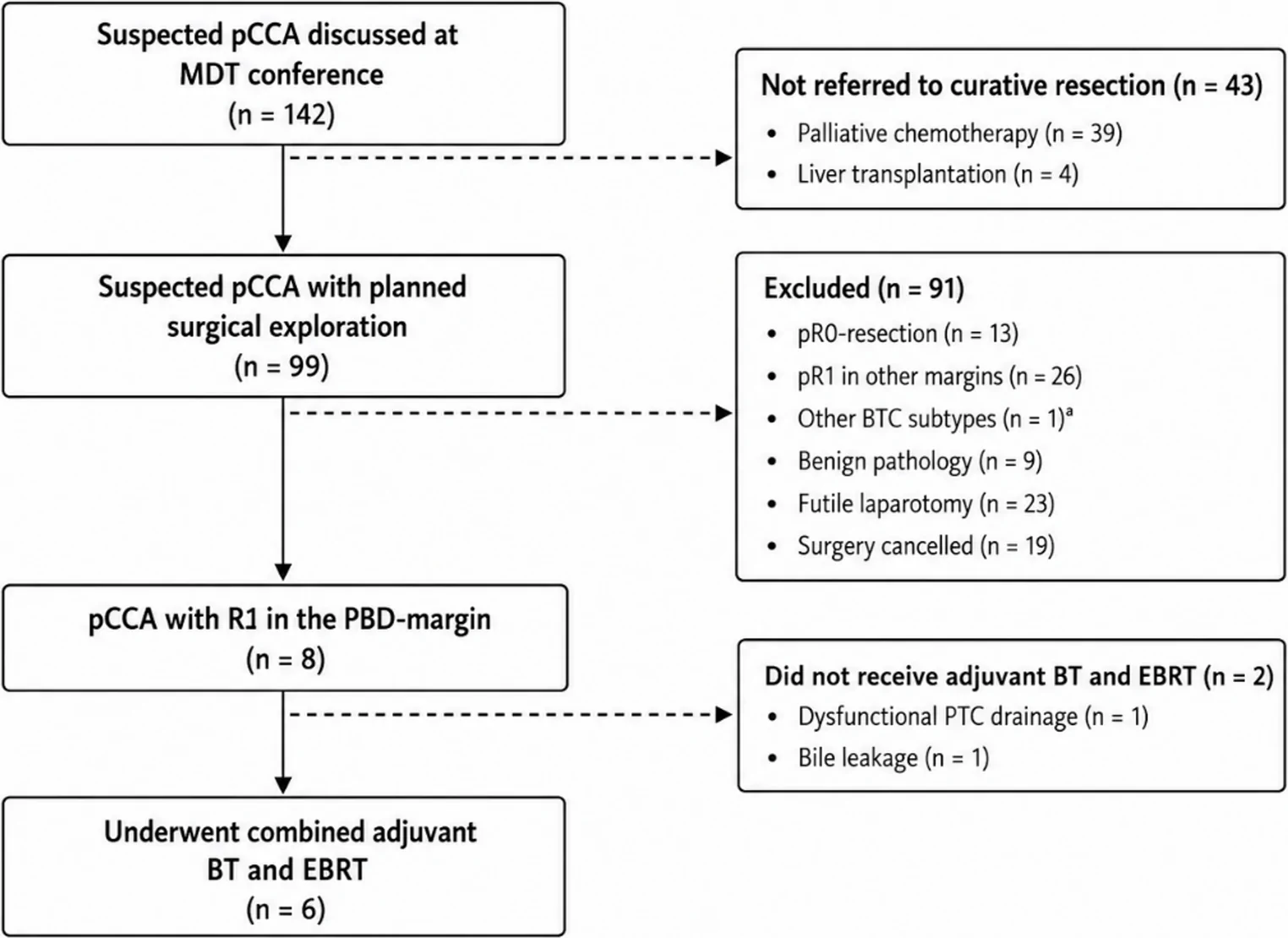
Flow diagram of patient selection and eligibility for treatment. Abbreviations: *BTC* biliary tract carcinoma, *PTC* percutaneous transhepatic cholangiography and drain, *PBD* proximal bile duct, *BT* brachytherapy, *EBRT* external beam radiation therapy. **a**: One with Intrahepatic Cholangiocarcinoma underwent combined *BT* and *EBRT* with chemotherapy due to R1 in the* PBD*

Between January 1, 2017, and December 31, 2021, 99 patients with suspected pCCA were planned for surgical exploration. Eight patients had pR1 only in PBD margin (8%) and were assessed at the MDT conference after resection, of whom 6 ultimately received combined BT and EBRT. Reasons for exclusion at each stage are shown in Fig. 1.

### Patient characteristics

The 6 treated patients had a median age of 57.6 years (IQR 56.4–71.5) and were all male with baseline characteristics presented in Table 1. Five of six patients underwent ERCP at local hospitals during diagnostic work-up prior to referral, and definitive PTC drainage. The median number of required biliary decompressions before resection was 2.5 (IQR 2–3), with complications including cholangitis (*n* = 3), pancreatitis (*n* = 1), or portal vein thrombosis (*n* = 1). One required PVE (*n* = 1). None received neoadjuvant chemotherapy. Median time from first MDT to resection was 63 days (IQR 37–71). Three underwent trisectionectomy; two left-sided (*n* = 2) and one right-sided (*n* = 1), all with segment 1 resection. The rest underwent extrahepatic bile duct resection without hepatic resection (*n* = 3), of whom one required concurrent pancreatoduodenectomy (PD). Intraoperative PBD FS analysis was undertaken in 4 patients, of which 3 remained positive despite re-resection, whereas 1 FS analysis yielded a false-negative result. No patient (*n* = 0) underwent vascular resection.

**Table 1:** Treatment, intraoperative complications and histopathology

Patient (*n* = 6)		1	2	3	4	5	6
Variable							
Age, years, median (IQR)	57.6 (56.4–71.5)	58	58	63	36	79	56
Sex	Descriptive	Male	Male	Male	Male	Male	Male
Comorbidities	Descriptive	Hypertension Gout Gastrointestinal reflux	None	Seized alcohol overconsumption without organ damage	Primary Sclerosing Cholangitis Ulcerative Cholitis	Gout Cardiac arrythmia with pacemaker	Ischemic stroke Hypertension Gout
ASA classification	Descriptive	II	I	II	III	III	III
ECOG-PS	Descriptive	0	0	0	1	1	1
BMI, kg/m², median (IQR)	26.7 (22.6–32.3)	25.8	26.6	21.6	18.0	36.5	34.3
Bismuth type	Descriptive	I	IV	II	II	I	IV
Portal vein invasion	Descriptive	No	No	No	No	No	Yes
Hepatic artery invasion	Descriptive	No	No	No	No	No	No
Biliary decompressions, n, median (IQR)	2.5 (2–3)	1	2	2	3	3	3
Decompression type	Descriptive	PTC	PTC and ERCP	PTC and ERCP	PTC and ERCP	PTC and ERCP	PTC and ERCP
PVE	Descriptive	No	No	No	No	No	Yes
Neoadjuvant chemotherapy	Descriptive	No	No	No	No	No	No
Bilirubin, µmol/L before drainage, median (IQR)	72.5 (19–83)	17	100	90	72	76	78
Bilirubin, µmol/L after drainage, median (IQR)	13 (11–20)	15	11	7	76	11	21
CA 19 − 9, u/L, median (IQR)	203 (158–332)	Missing	15	332	203	158	213
Time from MDT to surgery, days, median (IQR)	63 (37–71)	26	60	30	66	79	73
Resection type	Descriptive	Extrahepatic bile duct resection	LT	LT	Extrahepatic bile duct resection with PD	Extrahepatic bile duct resection	RT
Vascular reconstruction	Descriptive	No	No	No	No	No	No
PBD frozen section result	Descriptive	False negative	Not conducted	Positive at second re-resection	Positive at second re-resection	Positive at first re-resection	Not conducted
Blood loss, mL, median (IQR)	1632 (1109–2801)	1135	3025	900	2130	1100	3800
Operation time, min, median (IQR)	304 (243–342)	197	417	348	324	228	284
CCI, median (IQR)	52.3 (30.0-57.8)	47.7	56.8	24.2	67.4	24.2	58.1
Bile-leakage, ISGLS grade A to C	Descriptive	None	None	None	None	None	None
Intra-abdominal abscess	Descriptive	No	No	No	No	No	No
Liver failure, ISGLS grade A to C	Descriptive	No	No	No	No	No	B
Bilirubin at discharge, µmol/L, median (IQR)	7.5 (5.5–11.8)	8	5	7	13	4	37
Time to discharge, days, median (IQR)	19.5 (11.5–27.5)	6	23	10	42	16	29
TNM (AJCC)	Descriptive	T2aN0M0	T2aN0M0	T2aN0M0	T2bN1M0	T2bN1M0	T2bN1M0
R1 in other margins	Descriptive	No	No	No	No	No	Radial
Pn invasion	Descriptive	Yes	Yes	Yes	Yes	No	Yes
Lv invasion	Descriptive	No	Yes	Yes	Yes	No	No
Tumour differentiation	Descriptive	Moderate	Moderate	Moderate	Poor	Moderate	Moderate
Tumour size, mm, median (IQR)	15 (15–22)	7	22	15	15	23	30

Abbreviations: *ASA* American Society of Anesthesiologists,* ECOG -PS* Eastern Cooperative Oncology Group performance status, *BMI* body mass index, *PVE* portal vein embolization, *ERCP* endoscopic retrograde cholangiopancreatography, *PTC* percutaneous transhepatic cholangiography and drain, *MDT* multidisciplinary team, *RT* right trisectionectomy, *LT* left trisectionectomy, *PD* pancreatoduodenectomy, *PBD* proximal bile duct, *CCI* comprehensive complication index, *ISGLS* International Study Group of Liver Surgery, *AJCC* American Joint Committee on Cancer 8th edition, *Pn* perineural, *Lv* lymphovascular. *IQR* interquartile range. Continuous variables are presented as median (*IQR*)

Median CCI was 52.25 (IQR 30.0–57.8). Two patients (*n* = 2) developed fascia deadhesion, one (*n* = 1) developed ISGLS grade B liver failure [32], and no bile leakage was observed. Median bilirubin at discharge was 7.5 µmol/L (IQR 5.5–11.75). All patients (*n* = 6) had clinical stage T2a or T2b tumours, with half (*n* = 3) presenting with regional lymph node metastasis (N1). All patients (*n* = 6) had an isolated pR1 in PBD margin; however retrospective re-evaluation identified one case (*n* = 1) with pR1 in the circumferential margin.

The median time from resection to BT initiation was 39.5 days (IQR 34.3–52.3).

Adjuvant chemotherapy strategies varied: three patients received capecitabine, of which one switched to combined gemcitabine with cisplatin due to toxicity. One patient received combined capecitabine with gemcitabine. Two did not receive adjuvant chemotherapy owing to prolonged time from surgical resection and patient refusal, respectively.

### Safety

TRAEs are detailed in Table 2. Early TRAEs were reported in 5 of 6 patients, with most classified as grade 1 or 2 (*n* = 4), and two as grade 3; no early grade 4 or 5 TRAE was reported. Early TRAEs included cholangitis or nausea following BT, abdominal pain or paresthesia after EBRT. One patient developed a subcutaneous infection at the PTC site.

**Table 2:** Toxicity and oncological outcomes

Patient (*n* = 6)			1	2	3	4	5	6
Variable								
Time from resection to BT, days, median (IQR)	39.5 (34.3–52.3)		74	53	16	34	35	44
Early TRAEs	Descriptive		Cholangitis managed by IV antibiotics after BT	Paresthesia after BT	Subcutaneous infection from PTC drainage managed with oral antibiotics	Cholangitis managed by IV antibiotics after BT. Nausea managed by oral pantoprazole and ondansetron after EBRT.	Nausea and abdominal pain managed by oral morphine, pantoprazole and ondansetron after EBRT.	None
Radiation-induced biliary stricture	Descriptive		Yes	No	Yes	Yes	Yes	No
Time to radiation-induced biliary stricture, months, median (IQR)	30.4 (6.5–50.1)		30.4	NA	50.1	6.5	43.2	NA
Late TRAEs	Descriptive		Recurrent cholangitis requiring lV antibiotics. Liver abscess requiring IV antibiotics and drainage. Repeated admissions due to pyloric stenosis with fluid loss requiring a gastro-entero anastomosis Porto-biliary fistula with pseudo aneurism. Spontaneous bleeding from colon mesentery collateral with cardiac arrest.	None	Cholangitis requiring IV or oral antibiotics	Cholangitis requiring IV antibiotics	Cholangitis requiring IV antibiotics. Gastric antral vascular ectasia with blood transfusions and multiple argon beam treatments.	None
TRAEs Grade 1, n	1		0	1	0	0	0	0
TRAEs Grade 2, n	5		0	0	3	1	1	0
TRAEs Grade 3, n	15		5	0	3	2	5	0
TRAEs Grade 4, n	2		2	0	0	0	0	0
TRAEs Grade 5, n	1		0	0	0	0	1	0
TRAE-related readmissions, median (IQR)	3 (0.75–4.5)		6	0	3	3	5	0
PTC to relieve benign radiation-induced biliary stricture	Descriptive		Yes	NA	No	Yes	No	NA
Adjuvant chemotherapy	Descriptive		Combined capecitabine and gemcitabine	No	Capecitabine 3 cycles; switched to gemcitabine due to toxicity	Capecitabine	Capecitabine	No
Chemotherapy cycles, n, median (IQR)	6 (1.5–7.5)		6	0	6	8	8	0
Survival								
Follow up, months, median (IQR)	35.5 (21.1–61.8)		64.1	17.9	87.1	23.9	44.8	17.6
Recurrence	Descriptive		Liver and Lungs	Liver	None	Liver	None	Peritoneum and Lungs
DFS, months, median (IQR)	28.6 (10.9–53.5)		56.4	7.7	NA	12.4	44.8	10.4
Death	Descriptive		Yes	Yes	No	Yes	Yes	Yes
Cause of death	Descriptive		Disease related	Disease related	NA	Disease related	Cholangitis with septic shock	Disease related
OS, months, median (IQR)	34.3 (19.4–39.9)		64.1	17.9	NA	23.9	44.8	17.6

Abbreviations:* BT* brachytherapy,* EBRT* external beam radiotherapy, *TRAE* treatment-related adverse event, *PTC* percutaneous transhepatic cholangiography and drain, *DFS* disease-free survival, *OS* overall survival, *IQR* interquartile range, *NA* not applicable. Continuous variables are presented as median (*IQR*)

**Fig. 2:**
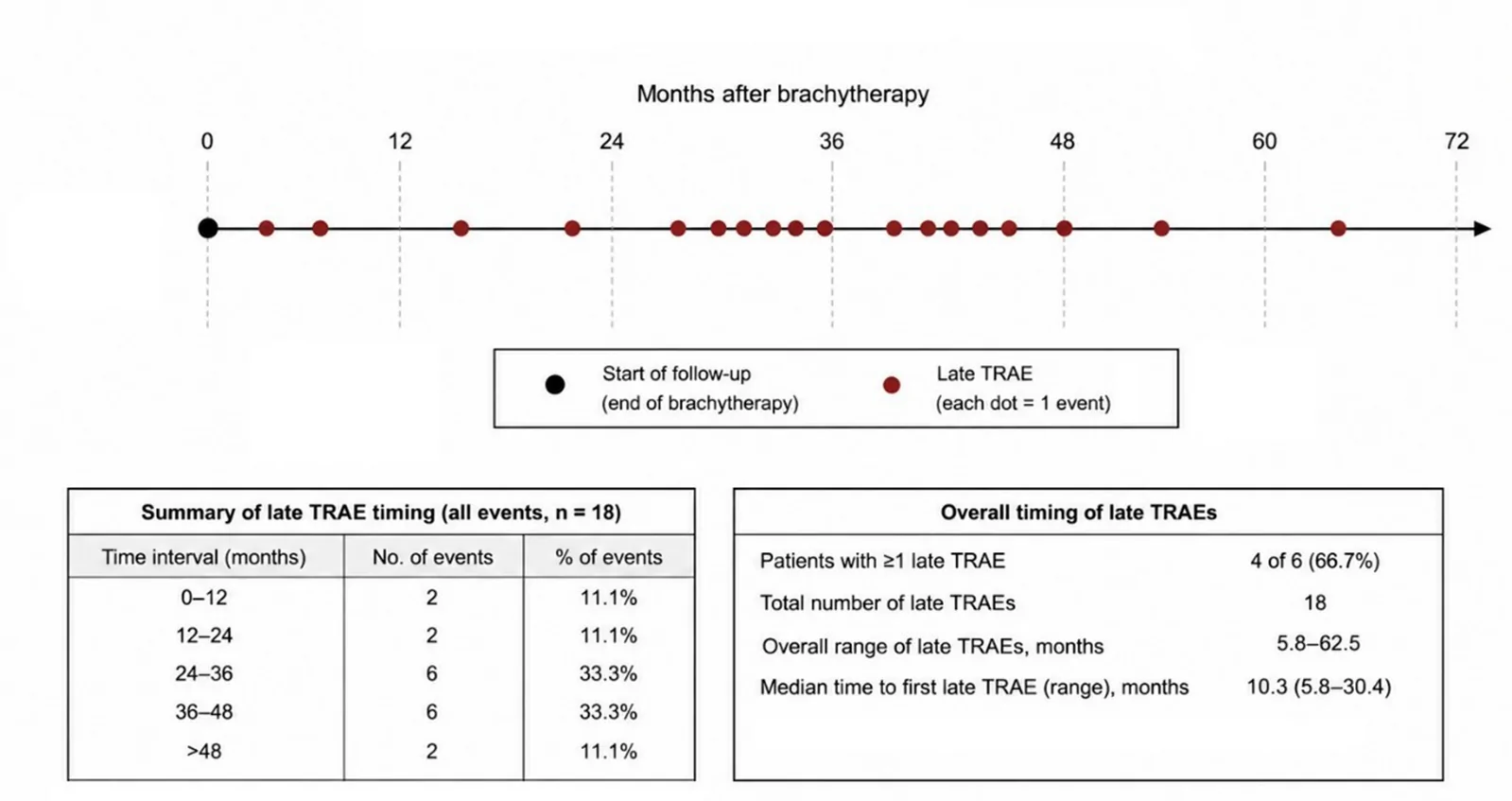
Distribution and timing of treatment related adverse events The earliest late TRAE occurred 5.8 months after brachytherapy, whereas the latest occurred 62.5 months after treatment. Two events occurred within the first year, two between 12 and 24 months, six between 24 and 36 months, six between 36 and 48 months, and two beyond 48 months

Four of six patients (66.7%) developed a total of 18 late TRAEs. All 4 affected patients developed radiation-induced benign biliary strictures, occurring at a median of 30.4 months (IQR 6.5–50.1) after BT. All 4 patients experienced severe late TRAEs (grade ≥ 3), with most classified as grade 3 (*n* = 12) or grade 4 (*n* = 3), and one patient dying after cholangitis with septic shock (grade 5). Two (*n* = 2) required biliary decompression. Most severe late TRAEs occurred after 2 years, and the distribution over time is illustrated in Fig. 2. Of note, only two benign hepaticojejunostomy strictures a matched cohort of patients with pR1 who did not receive BT/EBRT during the same period (*n* = 34), both occurring within 1 year after surgery (*Supplementary Table **1*).

Median TRAE-related re-admissions were 3 (IQR 0.75–4.50), mainly due to late TRAEs. Late TRAEs included recurrent cholangitis managed by IV or oral antibiotics, cholangitis with liver abscess, radiation-induced pyloric stenosis or gastric antral vascular ectasia, porto-biliary fistula, and spontaneous mesentery collateral bleeding requiring resuscitation after cardiac arrest.

### Follow up and survival

**Fig. 3:**
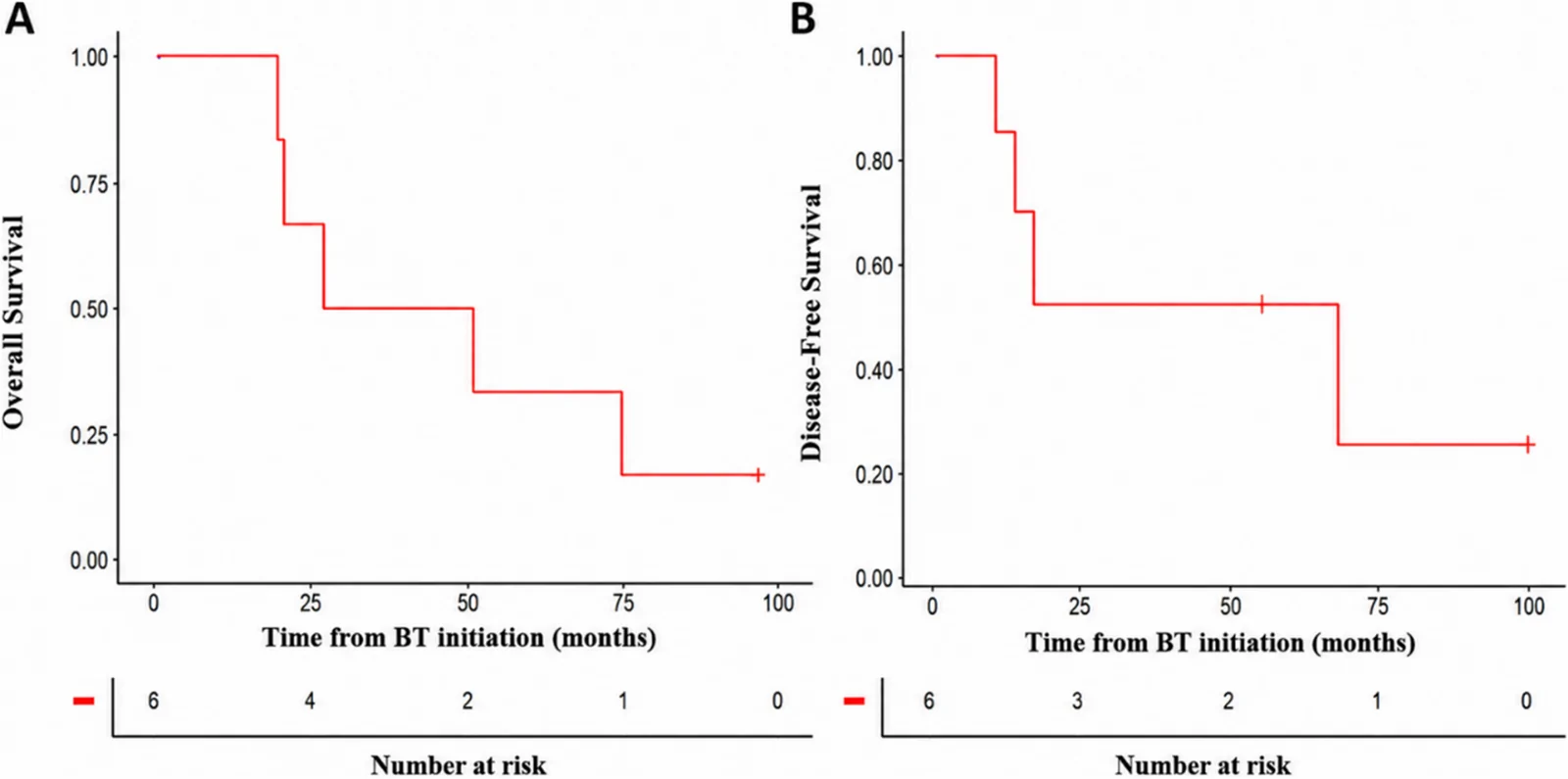
Overall (**A**) and disease-free survival (**B**) for patients (*n* = 6) following combined BT with EBRT

Survival data are presented in Table 2; Fig. 3. Survival analysis was performed 47 months after enrollment of the last patient. After a median follow up time of 35.5 months (IQR 21.1–61.8), 5 of 6 patients had died, 4 of whom developed recurrence. Median OS was 34.3 months (IQR 19.4–39.9) and median DFS was 28.6 months (IQR 10.9–53.5). Recurrence was either multifocal without local involvement (*n* = 2) or confined to the liver parenchyma (*n* = 3); local recurrences were not observed (*n* = 0).

## Discussion

This prospective registration study presents a contemporary report of combined adjuvant BT with EBRT for pR1 in the PBD margin for resected pCCA, including TRAEs, and survival. The results demonstrate a substantial incidence of late severe TRAEs.

Early TRAEs were infrequent, with few severe TRAEs. However, a substantial proportion of patients developed radiation-induced benign biliary strictures, resulting in late severe TRAEs dominated by recurrent cholangitis with readmissions, and PTC-interventions for infection control. This likely reflects the intrinsic radiosensitivity of biliary structures, predisposing to stricture formation which—unlike in the palliative setting—cannot be mitigated by pre-emptive permanent biliary decompression [15–17]. Interestingly, three of four patients developed radiation-induced biliary strictures at least 2.5 years after completing BT, with most late TRAEs occurring more than 2 years after treatment. This underscores the need for long-term follow up beyond the end of inclusion period for adequate assessment of late toxicity. To date, late radiation-induced toxicity in patients with resected pCCA treated with combined BT and EBRT has only been described by *Gerhards et al.* nearly two decades ago [19]. Notably, rates of late TRAEs in the present contemporary cohort were comparable, emphasizing the continued importance of careful patient selection.

Unlike our study, earlier cohorts investigating this approach were limited by non-standardized patient selection [19, 21], lack of pathological stratification [30], and the use of outdated radiotherapy techniques [19, 21]. Despite the small patient numbers undergoing BT with EBRT (*n* = 6), the observed DFS of 28.6 months and OS of 34.3 months are only slightly lower than the previously reported survival for R0 patients in our cohort (39.9 and 30.6 months, respectively) [29]. While four out of six patients experienced recurrence, none recurred locally— implying that the treatment may offer local disease control. The findings align with historical reports of adjuvant BT with EBRT that suggested reduced local recurrence rates for pCCA [36]. However, any benefit by locoregional control should be weighed against the observed TRAE-related morbidity and mortality. Also, the majority in the present study still recurred with distant metastasis, questioning the ability to achieve systemic disease control by combined BT with EBRT.

This study has several limitations. First, the small sample size combined with selection bias limits generalizability, warranting larger prospective and multicentre cohorts. Second, while postoperative benign biliary strictures without prior radiotherapy are uncommon in pCCA, some cases may have represented undetected local recurrence [37]. Although local recurrence was assessed using needle biopsy or cholangiography-guided SpyBites, obtaining representative material from extrahepatic biliary strictures remains difficult [38]. However, the incidence of benign hepaticojejunostomy strictures was substantially lower in the matched pR1 cohort without combined EBRT and BT. Together with the long follow up duration in the present study, this suggests that the strictures observed following combined EBRT and BT were more likely treatment-related rather than manifestations of local recurrence. Third, no firm conclusions can be drawn regarding whether BT alone, EBRT alone, or their combination was responsible for the observed high rates of radiation-induced biliary strictures and subsequent late TRAEs. Fourth, heterogeneity in chemotherapeutic regimens, and incomplete delivery, may confound interpretation of systemic versus local treatment effects of combined BT with EBRT. However, all patients who initiated adjuvant chemotherapy received capecitabine and-/or gemcitabine— either in combination or sequentially—regimens with limited documented effect in resected pCCA [12, 39]. Fifth, the impact of BT alone or with EBRT on survival, remains unclear within our selection criteria, especially as older limited retrospective data show no clear benefit from adding BT [19]. Lastly, lymph node metastases were present in half of the cohort (*n* = 3), which may have contributed to high rates of distal recurrence. This is highlighted by recent findings suggesting that importance of pR0 diminishes in the presence of lymph node metastasis [40]. Accordingly, any future protocols should exclude patients with lymph node metastasis, as local treatment targeting isolated pR1 at the PBD is unlikely to provide meaningful oncologic benefit while exposing patient to late treatment-related toxicity.

Future studies should therefore approach combined adjuvant BT with EBRT with caution and account for adverse histopathological factors including recent findings concerning margin specific recurrence patterns [36, 41]. Prospective evaluation with predefined safety endpoints is also warranted prior to routine use. Furthermore, evaluation of quality-of-life outcomes and biliary complications is essential to balance survival benefit against late morbidity. While a randomized controlled trial would overcome most limitations, this is not possible due to the small pool of eligible patients. Higher-quality evidence will likely depend on international multicenter collaboration among high-volume HPB centers to further evaluate the role of combined BT and EBRT in resected pCCA.

## Conclusion

In summary, while combined BT with EBRT may offer local disease control for pCCA in PBD pR1, the high burden of late severe TRAEs warrants careful consideration of its overall benefit. Based on the available evidence, we do not recommend its use outside clinical trials.

## Supplementary information

Below is the link to the electronic supplementary material.

Supplementary Material 1

## Data Availability

The data sets from the present study are not publicly available owing to restrictions on ethical approval from the Danish Health Authority, Capital Region of Denmark (R-23057752).

## Abbreviations

BT: brachytherapy
EBRT: external beam radiotherapy
pCCA: perihilar cholangiocarcinoma
PBD: proximal bile duct
DBD: distal bile duct
RM: radial margin
TRAE: treatment-related adverse event
OS: overall survival
DFS: disease-free survival
MDT: multidisciplinary team
FLR: future liver remnant
FS: frozen section
PTC: percutaneous transhepatic cholangiography
PVE: portal vein embolization
ECOG-PS: Eastern Cooperative Oncology Group performance status
ASA: American Society of Anesthesiologists
BMI: body mass index
CCI: comprehensive complication index
IQR: interquartile range
CTCAE: Common Terminology Criteria for Adverse Events
