# Supplementary material for: Safety of adjuvant brachytherapy combined with external beam radiotherapy for proximal bile duct pR1 in perihilar cholangiocarcinoma: a prospective registration study
Source: Langenbecks Arch Surg. 2026 Aug 5;411(1):302. doi: 10.1007/s00423-026-04180-6 (PMC13727926; doi:10.1007/s00423-026-04180-6)
Supplement: Supplementary file 1 [file 423_2026_4180_MOESM1_ESM.docx]

**Table 1.** Clinicopathological characteristics

| **Variable** |  |  |  |
| --- | --- | --- | --- |
|  | ***EBRT and BT* (n=6)** | ***R1 without EBRT and BT (n=34)*** | ***P* value** |
| Age, years, median (IQR) | 57.6 (56.4–71.5) | 66.8 (55.4–73.2) | 0.011^‡^ |
| Sex  Male  Female | 6 (100)  0 (0.0) | 14  15 | 0.019^†^ |
| ASA classification  I  II  III | 0 (0.0)  4 (66.7)  2 (33.3) | 1 (2.9)  20 (58.8)  13 (38.2) | 0.877^†^ |
| ECOG-PS  0  1 | 3 (50.0)  3 (50.0) | 19 (55.9)  15 (44.1) | 0.789^†^ |
| BMI, kg/m², median (IQR) | 26.7 (22.6–32.3) | 25.7 (24.1–28.3) | 0.370^‡^ |
| Bismuth–Corlette type  I–II  IIIa  IIIb  IV | 4 (66.7)  2 (33.3)  0 (0.0)  0 (0.0) | 9 (26.5)  11 (32.4)  5 (14.7)  9 (26.6) | 0.171^†^ |
| Decompression type (%)  None  ERCP  PTC  ERCP and PTC | 0 (0.0)  0 (0.0)  1 (16.7)  5 (83.3) | 1 (2.9)  1 (2.9)  11 (32.4)  21 (61.8) | 0.768^†^ |
| PVE  Yes  No | 1 (16.7)  5 (83.3) | 9 ()  25 () | 0.609^†^ |
| Neoadjuvant chemotherapy  Yes  No | 0 (0.0)  6 (100) | 4  30 | 0.375^†^ |
| Bilirubin, μmol/L after drainage, median (IQR) | 13.0 (11.0–20.0) | 20.0 (7.0-37.3) | 0.804^‡^ |
| Resection type  EHBDR  RT  LT | 3  1  2 | 5  8  16 | 0.220^†^ |
| Additional PD  Yes  No | 1 (16.7)  5 (83.3) | 1  33 | 0.670^†^ |
| Vascular reconstruction  Yes  No | 0 (0)  6 (100) | 4  30 | 0.375^†^ |
| CCI, median (IQR) | 52.3 (30.0–57.8) | 40.0 (23.4-57.0) | 0.448^‡^ |
| Bile-leakage ISGLS grade  None  Grade A  Grade B  Grade C | 6 (100)  0 (0.0)  0 (0.0)  0 (0.0) | 23  1  10  0 | 0.444^†^ |
| Intra-abdominal abscess  Yes  No | 0 (0)  6 (100) | 9  25 | 0.152^†^ |
| Liver failure ISGLS grade  None  Grade A  Grade B  Grade C | 5 (83.3%)  0 (0.0)  1 (16.7%)  0 (0.0) | 29 (85.3)  3 (8.8)  2 (5.9  0 (0.0) | 0.723^†^ |
| T-stage (AJCC)  T1  T2a  T2b  T3  T4 | 0 (0.0)  3 (50.0)  3 (50.0)  0 (0.0)  0 (0.0) | 0 (0.0)  9 (26.5)  20 (58.8)  3 (8.8)  3 (8.8) | 0.716^†^ |
| Lymph node metastasis  Yes  No | 3 (50.0)  3 (50.0) | 12 (35.3)  22 (64.7) | 0.492^†^ |
| Margin-specific involvement, n  Distal  Proximal  Combined proximal and radial  Radial | 0  5  1  0 | 0  0  6  28 | NA |
| Perineural invasion  Yes  No | 5  1 | 32  2 | 0.355^†^ |
| Tumour differentiation^a^  Well  Moderate  Poor | 0 (0.0)  5  1 | 6  20  7 | 0.460^†^ |
| Tumour size, mm, median (IQR) | 15.0 (15.0–22.0) | 22.5 (17.8–28.5) | 0.286^‡^ |
| Postoperative non-malignant biliary stricture  Yes  No | 4 (66.6)  2 (33.3) | 2^b^ (5.9)  32 (94.1) | <0.001^†^ |
| Other hepaticojejunostomy related morbidity, descriptive | Pyloric stenosis  Porto-biliary fistula  Gastric vascular ectasia | Bile fistula^c^  Bowl perforation at hepaticojejunostomy^c^ |  |
| Recurrence location  Local  Liver  Distant  Multifocal  None | 0  2  0  2  2 | 5  5  8  3  12 | 0.239^†^ |
| Death  Yes  No | 5  1 | 22 (64.7)  12 (35.3) | 0.369^†^ |
| 90-day mortality  Yes  No | 0 (0.0)  6 (100) | 1  33 | 0.670^†^ |
| OS, months, median (IQR) | 34.3 (19.4–39.9)^d^ | 29.9 (19.0-58.3) | 0.777^‡^ |
| DFS, months, median (IQR) | 28.6 (10.9–53.5)^d^ | 13.6 (8.9-42.7) | 0.756^‡^ |

**Supplementary Table 1.** Abbreviations**:** ASA, American Society of Anesthesiologists; ECOG-PS, Eastern Cooperative Oncology Group performance status; BMI, body mass index; PVE, portal vein embolisation; ERCP, endoscopic retrograde cholangiopancreatography; PTC, percutaneous transhepatic cholangiography; MDT, multidisciplinary team; EHBDR, extrahepatic bile duct resection; RT, right trisectionectomy; LT, left trisectionectomy; PD, pancreatoduodenectomy; CCI, comprehensive complication index; ISGLS, International Study Group of Liver Surgery; AJCC, American Joint Committee on Cancer 8th edition; IQR, interquartile rang; NA, not applicable. †: Chi-Square test. a, one patient missing. b, time to development of non-malignant biliary stricture at 9.6 and 2.6 months respectively. c, time to occurrence of other hepaticojejunostomy-related morbidity at 0.8 and 3.3 months respectively. d, patients at matched group with mortality <90 days excluded (n=1) to address immortal time bias. Variables described as n (%) unless stated otherwise. ‡: Mann-Whitney U test. Significance at *P*<0.050.
